# Abscess of the Tonsils

**Published:** 1879-05

**Authors:** 


Abscess of the Tonsils.—According to M. Verneuil the purulent collection whose formation is the termination of an inflammation of the tonsil is not situated in the substance of the tonsil, but outside the gland, in the connective tissue that separates it from the bottom of the fossa in which it is lodged. The tonsil is very loosely attached to the walls of its fossa. Becoming tumefied under the influence of the inflammation, it projects between the pillars of the fauces; and at each movement of deglutition there may be seen, thanks to this feeble attachment, slight to and fro movements from before backwards, which may easily be proved by inspection of the throat. This mobility is not without its influence in the production of the abscess. In consequence of the continual displacement of the gland, there is formed outside the tonsil a serous bursa in the connective tissue that extends from the one pillar to the other, and fills up the tonsillar fossa. It is in this bursa that the purulent collection is developed. The abscess is always very deeply situated, and it is with much difficulty reached with the knife. An incision made upon the swelling that the tonsil forms in the isthmus of the fauces has no chance of reaching it. To open it one must go through the anterior pillar of the fauces; it is this pillar that, elongated and pressed forward, forms the anterior wall of the abscess; but the anterior pillar is always œdematous, and to pass through it a very deep incision must be made—an incision that one dares not make sufficiently free on account of the fear of reaching the carotid artery. The rule that M. Verneuil's investigations have led him to lay down, is to leave these so-called abscesses of the tonsil to follow out their natural course. No effort should be made to open them; the pus should be allowed to spontaneously make its way through the anterior pillar. This spontaneous termination of the affection does not require very much time; on the fourth or fifth day the abscess opens of itself.—*Gaz. des Hopitaux.*

